# Utilizing Life's Crucial 9 for Rheumatoid Arthritis Risk Prediction: A Machine Learning Approach Based on NHANES Data

**DOI:** 10.1002/fsn3.71931

**Published:** 2026-05-21

**Authors:** Jiasi Zheng, Yuanyuan Gao, Xuemei Yuan, Feng Luo, Zhen Wang, Ziyuan Song, Wukai Ma

**Affiliations:** ^1^ Guizhou University of Traditional Chinese Medicine Guiyang China; ^2^ The Second Affiliated Hospital of Guizhou University of Traditional Chinese Medicine Guiyang China

**Keywords:** cardiovascular, machine learning, NHANES, rheumatoid arthritis, risk assessment

## Abstract

To investigate the association between the cardiovascular health metric Life's Crucial 9 (LC9) and the risk of rheumatoid arthritis (RA), and to establish a predictive model for RA risk. Data were obtained from the U.S. National Health and Nutrition Examination Survey (NHANES) 2011–2018, including 16,154 adults aged ≥ 20 years. RA status was determined by self‐report (RA group: 836 cases; non‐RA group: 15,318 cases). Weighted multivariable logistic regression was used to analyze the association between LC9 score (as both a continuous variable and quartiles) and RA, with restricted cubic splines applied to test for nonlinearity. LC9 score together with 10 covariates (age, sex, race, education, marital status, PIR group, AST, ALT, HDL cholesterol, and alcohol consumption) were incorporated into multiple machine learning algorithms (random forest, support vector machine, K‐nearest neighbor, XGBoost, LightGBM, and naive Bayes) to construct prediction models. The dataset was randomly divided into training and testing sets in a 7:3 ratio, with five‐fold cross‐validation for parameter optimization. Each 1‐unit increase in LC9 score was associated with a 27% reduction in RA risk (OR = 0.73, 95% CI: 0.68–0.80, *p* < 0.0001). Compared with the lowest quartile (Q1), the highest quartile (Q4) had a 57% lower risk (OR = 0.43, 95% CI: 0.33–0.55, *p* < 0.0001). A linear dose–response relationship was observed (P for trend < 0.0001; P for nonlinearity = 0.159). Among machine learning models, XGBoost performed best (test set AUC = 0.987, accuracy = 96.20%, sensitivity = 95.06%), followed by LightGBM (AUC = 0.982). Random forest (AUC = 0.817) and naive Bayes (AUC = 0.735) showed relatively weaker performance. SHAP analysis indicated that AST, age, ALT, and HDL were the top four predictors, while LC9, alcohol consumption, and PIR group had comparable importance, all significantly higher than other variables. LC9 is an independent protective factor against RA risk. An XGBoost‐based predictive model integrating LC9 demonstrated excellent diagnostic performance and may serve as a valuable tool for risk stratification in high‐risk RA populations.

## Graphical abstract

This study based on NHANES 2011–2018 (16,154 adults) found that higher LC9 score is an independent protective factor for rheumatoid arthritis (RA), with each 1‐unit increase associated with a 27% lower risk (OR = 0.73). An XGBoost machine learning model integrating LC9 and clinical covariates achieved excellent predictive performance (test AUC = 0.987). LC9 is a quantifiable, modifiable risk stratification tool for high‐risk RA populations.

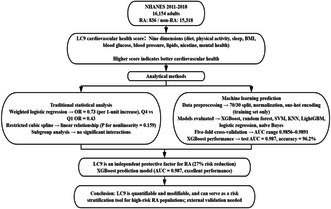

**Abbreviations**
ABSI: A Body Shape Index
ALT: Alanine Aminotransferase
AST: Aspartate Aminotransferase
AUC: Area Under the Curve
BMI: Body Mass Index
CAL: Clinical Attachment Loss
CI: Confidence Interval
CVH: Cardiovascular Health
DBP: Diastolic Blood Pressure
FPG: Fasting Plasma Glucose
HDL: High‐Density Lipoprotein Cholesterol
LC9: Life's Crucial 9
LDL: Low‐Density Lipoprotein Cholesterol
LE8: Life's Essential 8
MCC: Matthews Correlation Coefficient
NHANES: National Health and Nutrition Examination Survey
OR: Odds Ratio
PD: Pocket Depth
PHQ‐9: Patient Health Questionnaire‐9
PIR: Poverty Income Ratio
RA: Rheumatoid Arthritis
RCS: Restricted Cubic Spline
ROC: Receiver Operating Characteristic
SBP: Systolic Blood Pressure
SHAP: SHapley Additive exPlanations
SII: Systemic Immune‐Inflammation Index
SIRI: Systemic Inflammation Response Index
TC: Total Cholesterol
TG: Triglycerides
TNF‐α: Tumor Necrosis Factor‐alpha
WC: Waist Circumference
WHtR: Waist‐to‐Height Ratio
WWI: Weight‐adjusted Waist Index
XGBoost: eXtreme Gradient Boosting

## 1 Introduction

Rheumatoid arthritis (RA) is a systemic autoimmune disease characterized by chronic synovial inflammation and progressive joint destruction (Bottini and Firestein 2013; Kawaguchi et al. 2018). According to recent epidemiological data, the global prevalence of RA has reached 0.27% in recent years, with the incidence in women being approximately twice that in men (Finckh et al. 2022; Lin et al. 2020). In addition to causing joint deformity and functional loss, RA significantly increases the risk of cardiovascular events, which has become the leading cause of long‐term mortality among patients (Drosos et al. 2024). Pathological and imaging studies consistently demonstrate a higher burden of coronary atherosclerotic plaques in RA patients compared with healthy individuals (Popova et al. 2022; Patel et al. 2022). The underlying mechanism involves chronic inflammation‐induced systemic vascular endothelial injury (Khanna et al. 2019). Persistently elevated levels of serum tumor necrosis factor‐α (TNF‐α) and interleukin‐6 (IL‐6) promote the expression of endothelial adhesion molecules, accelerate monocyte infiltration, and facilitate the formation of atherosclerotic plaques (Popova et al. 2022). Meanwhile, immune complexes mediated by autoantibodies deposit within the vascular wall and trigger vascular inflammatory lesions through complement cascade activation (Hysa et al. 2021). Evidence indicates that immune‐cardiovascular comorbidity is particularly pronounced in RA patients, who face a 2‐ to 3‐fold higher risk of myocardial infarction, heart failure, and sudden cardiac death compared with non‐RA populations (Liao and Solomon 2013).

In this context, the early identification of risk factors for cardiovascular events in patients with RA, along with the implementation of targeted interventions, holds significant importance for reducing the incidence of such events. In 2022, the American Heart Association introduced the “Life's Crucial 9 (LC9)” metrics, offering a novel perspective for quantifying cardiometabolic‐immune homeostasis (Lloyd‐Jones et al. 2022). A growing body of research has revealed that several components of LC9 are directly related to RA pathogenesis. For example, leptin secreted by obese adipose tissue can activate synovial macrophages to release interleukin‐8 (IL‐8) (Song et al. 2020); hyperglycemia enhances osteoclast activity through the receptor for advanced glycation end products (RAGE) pathway (Cui et al. 2011); and psychological stress‐induced glucocorticoid resistance may aggravate systemic inflammatory responses (Sharif et al. 2018). Therefore, the LC9 score may serve as a bridging indicator that links RA disease activity with cardiovascular risk.

Based on data from the National Health and Nutrition Examination Survey (NHANES), this study is the first to investigate the association between the LC9 score and RA risk, and to construct a machine learning prediction model. By integrating multidimensional health data with advanced algorithms, this work aims to provide new strategies for the early prevention of RA and its cardiovascular complications, thereby filling the theoretical gap in systematic health interventions within the current diagnostic and treatment framework.

## 2 Materials and Methods

### 2.1 Data Source

This study leveraged data from the National Health and Nutrition Examination Survey (NHANES), a nationally representative program administered by the U.S. Centers for Disease Control and Prevention, renowned for its rigorous quality control and open data access (https://www.cdc.gov/nchs/nhanes). Four consecutive NHANES cycles from 2011 to 2018 were included for analysis, targeting adults aged ≥ 20 years. RA status was identified via self‐reported responses in the Medical Conditions Questionnaire, and the LC9 score was derived from corresponding variables available in each survey wave.

A total of 39,156 participants were initially screened. We excluded individuals younger than 20 years (*n* = 16,539), those missing RA outcome data (*n* = 51), those lacking information on LC9 component variables (*n* = 6355), and those with extreme LC9 outliers (*n* = 57). The final analytic sample comprised 16,154 participants (Zhu et al. 2025).

### 2.2 Self‐Reported Disease Status

Rheumatoid arthritis (RA) status (Matsunaga et al. 2021) was determined based on responses to the Medical Conditions Questionnaire. Participants who self‐reported having been diagnosed with “rheumatoid arthritis” by a doctor or other health professional were classified as having RA. Those who reported no history of arthritis or other types of arthritis were excluded from the analysis.

### 2.3 Definition of LC9

LC9 is a comprehensive cardiovascular health (CVH) metric proposed by the American Heart Association (AHA) in 2022. It integrates the original eight components of Life's Essential 8 (LE8)–including diet, physical activity, nicotine exposure, sleep, body mass index (BMI), non‐high‐density lipoprotein cholesterol (non‐HDL‐C), blood glucose, and blood pressure–with an additional dimension of mental health, assessed via the Patient Health Questionnaire‐9 (PHQ‐9) (Zhu et al. 2025).

We constructed the LC9 score based on NHANES data. Each of the nine components was scored on a continuous scale from 0 to 100. A valid LC9 score was computed as the arithmetic mean of the available component scores, provided that data were complete for at least eight components. Higher LC9 scores indicate better overall cardiovascular and psychological health, reflecting closer alignment with ideal health status. For analytical purposes, the LC9 score was further categorized into quartiles (LC9_Equal Size Bin), with categories 1–4 denoting progressively higher levels of cardiovascular health.

### 2.4 Covariates

The covariates included in this analysis were: age (continuous, years), sex (male/female), race/ethnicity (non‐Hispanic White, non‐Hispanic Black, Mexican American, and other), educational attainment (<high school, high school, >high school), marital status (married, never married, other), and poverty income ratio (PIR, continuous). Given the potential impact of alcohol consumption on the risk of RA, self‐reported drinking behavior was also included as a covariate and classified into three categories: never, occasional, and frequent drinking. All covariates were defined and coded based on the official NHANES documentation. Categorical variables were incorporated into regression models using dummy variables, while continuous variables were included in their original scale for statistical analysis (Zhu et al. 2025).

### 2.5 Machine Learning Modeling

Machine learning–based classification models were developed using Python (v3.10) to predict RA status. The initial dataset comprised both continuous and categorical variables. Continuous features were standardized using *Z*‐score normalization, and categorical variables were encoded using one‐hot encoding. These preprocessing steps were fitted exclusively on the training set and then applied to the test set to avoid data leakage. To ensure model robustness, the dataset was randomly partitioned into training (70%) and testing (30%) subsets, with five‐fold cross‐validation used on the training set for model selection and hyperparameter tuning.

Model development was conducted within the Scikit‐learn and XGBoost frameworks, incorporating multiple widely used classification algorithms, including logistic regression, random forest (RF), extreme gradient boosting (XGBoost), support vector machines (SVM), as well as K‐nearest neighbors (KNN) and Light Gradient Boosting Machine (LightGBM), and naïve Bayes. RA status was treated as the binary outcome variable. Predictor variables included LC9 scores and ten covariates: age, sex, race/ethnicity, education level, marital status, PIR group, AST, ALT, HDL cholesterol, and alcohol consumption.

Model performance was evaluated based on multiple metrics, including accuracy, F1 score, precision, recall, specificity, Matthews correlation coefficient (MCC), and the area under the receiver operating characteristic curve (AUC). To enhance interpretability, we employed SHapley Additive exPlanations (SHAP) to quantify the global and local contribution of each feature to model predictions, thereby elucidating the direction and magnitude of key variable influences (Qi et al. 2025).

### 2.6 Statistical Analysis

Multivariable binary logistic regression models were applied to examine the association between LC9 scores and RA. LC9 was modeled both as a categorical variable based on quartiles and as a continuous variable. Odds ratios (ORs) and corresponding 95% confidence intervals (CIs) were reported. For the quartile‐based analysis, the lowest quartile (Q1) served as the reference group. In the continuous model, LC9 was treated as a linear term, and a P for trend was calculated using the median value of each quartile to assess linearity across categories (Matsunaga et al. 2021).

To explore potential nonlinear associations between LC9 and RA, restricted cubic spline (RCS) logistic regression was employed, with LC9 modeled as a continuous variable using five predefined knots (at 43.89, 60.00, 68.89, 77.22, and 90.56), and 28.89 set as the reference point. Covariates adjusted in the model included sex, age, race/ethnicity, education, marital status, PIR grouping, AST, ALT, HDL, smoking status, and alcohol use. Results were expressed as ORs with 95% CIs (Wang et al. 2025).

Subgroup analyses were conducted using univariate generalized linear models (GLMs) with a binomial distribution, with RA as the outcome and LC9 as the independent variable. Stratified analyses were performed across sex, alcohol consumption status, PIR group, and marital status. In each subgroup model, covariates including age, race/ethnicity, education, AST, ALT, and HDL were adjusted. Interaction terms were assessed using P for interaction, and statistical significance was defined as a two‐tailed *p*‐value < 0.05 (Volpe et al. 2018).

All machine learning analyses were conducted in Python using libraries including xgboost, scikit‐learn, shap, pandas, and matplotlib. Conventional statistical analyses were performed in R (v4.4.1), utilizing packages such as survey, ggplot2, dplyr, mgcv, and rms. Two‐tailed statistical tests were applied, and *p* < 0.05 were considered statistically significant.

## 3 Result

### 3.1 Participant Characteristics

A total of 16,154 participants from the NHANES 2011–2018 cycles were included in this study, classified into two groups based on RA status: the RA group (*n* = 836) and the non‐RA group (*n* = 15,318), corresponding to a weighted national population of 182,198,287 individuals (Table 1; Table S1). Table 1 presents the weighted baseline characteristics. Compared with the non‐RA group, individuals with RA were significantly older on average (57.98 vs. 48.09 years) and had a higher proportion of women (57.88% vs. 51.86%). The RA group also exhibited elevated levels of body mass index (BMI: 30.71 vs. 29.38), systolic blood pressure (SBP: 126.28 vs. 122.18 mmHg), and fasting plasma glucose (FPG: 112.27 vs. 109.19 mg/dL). In terms of socioeconomic status, the RA group had a lower mean poverty income ratio (PIR: 2.55 vs. 3.04), with a higher proportion of individuals living below the poverty threshold (PIR < 1.30: 20.28% vs. 12.38%). The composite cardiovascular and psychosocial health score (LC9) was markedly lower in the RA group (63.38 vs. 70.31), suggesting poorer overall health status. The prevalence of comorbid conditions was significantly higher in the RA group, including hypertension (53.99% vs. 32.60%), dyslipidemia (50.60% vs. 34.92%), and diabetes (20.60% vs. 10.70%). Lifestyle patterns also differed between groups: participants with RA were less likely to engage in physical activity (38.71% vs. 47.78%), more likely to report smoking (55.69% vs. 42.70%), and slightly less likely to report alcohol consumption (70.12% vs. 76.64%). These patterns remained consistent in the unweighted analysis (Table S1), supporting the robustness of the findings.

**TABLE 1:** Weighted descriptive statistics based on NHANES survey design.

Characteristic	*N*	Overall *N* = 182,198,287	RA		*p*
			No *N* = 175,008,524	Yes *N* = 7,189,763	
Age	182,198,287	48.48 (17.00)	48.09 (17.01)	57.98 (13.57)	< 0.001
PIR	182,198,287	3.02 (1.59)	3.04 (1.59)	2.55 (1.56)	< 0.001
BMI	182,198,287	29.44 (6.98)	29.38 (6.96)	30.71 (7.47)	< 0.001
SBP	182,198,287	122.34 (15.94)	122.18 (15.87)	126.28 (17.09)	< 0.001
DBP	182,198,287	70.62 (11.10)	70.65 (10.99)	69.92 (13.49)	0.667
AST	182,198,287	24.76 (15.64)	24.77 (15.75)	24.58 (12.69)	0.995
ALT	182,198,287	24.71 (19.50)	24.74 (19.69)	24.13 (14.16)	0.656
FPG	182,198,287	109.32 (21.41)	109.19 (21.23)	112.27 (25.28)	0.044
HDL	182,198,287	53.88 (16.20)	53.88 (16.19)	53.96 (16.46)	0.986
TC	182,198,287	192.22 (40.99)	192.12 (40.97)	194.69 (41.27)	0.160
TG	182,198,287	119.45 (67.62)	119.30 (67.99)	123.14 (57.81)	0.330
LDL	182,198,287	112.60 (23.94)	112.58 (23.88)	112.97 (25.29)	0.879
LC9	182,198,287	70.04 (13.91)	70.31 (13.83)	63.38 (14.15)	< 0.001
Gender, *n* (*p*%)	182,198,287				0.037
Male		87,284,031 (47.91%)	84,255,565 (48.14%)	3,028,466 (42.12%)	
Female		94,914,255 (52.09%)	90,752,959 (51.86%)	4,161,297 (57.88%)	
Race, *n* (*p*%)	182,198,287				0.011
Mexican American		14,255,164 (7.82%)	13,731,661 (7.85%)	523,503 (7.28%)	
Non‐Hispanic Black		10,557,279 (5.79%)	10,158,658 (5.80%)	398,621 (5.54%)	
Non‐Hispanic White		123,201,472 (67.62%)	118,580,960 (67.76%)	4,620,512 (64.27%)	
Other Hispanic		19,893,305 (10.92%)	18,742,084 (10.71%)	1,151,221 (16.01%)	
Other race		14,291,066 (7.84%)	13,795,161 (7.88%)	495,905 (6.90%)	
Education, *n* (*p*%)	182,198,287				< 0.001
Above high school		15,748,066 (8.64%)	14,839,175 (8.48%)	908,891 (12.64%)	
High school		41,042,119 (22.53%)	39,150,574 (22.37%)	1,891,545 (26.31%)	
Under high school		125,408,102 (68.83%)	121,018,775 (69.15%)	4,389,327 (61.05%)	
Marital, *n* (*p*%)	182,198,287				0.469
Living alone		80,789,666 (44.34%)	77,727,249 (44.41%)	3,062,417 (42.59%)	
Living with a partner		101,408,620 (55.66%)	97,281,275 (55.59%)	4,127,345 (57.41%)	
PIR_Group, *n* (*p*%)	182,198,287				< 0.001
< 1.30		23,125,256 (12.69%)	21,667,261 (12.38%)	1,457,995 (20.28%)	
1.30 ~ 3.49		95,012,138 (52.15%)	90,989,596 (51.99%)	4,022,542 (55.95%)	
≥ 3.50		64,060,893 (35.16%)	62,351,668 (35.63%)	1,709,225 (23.77%)	
BMI_Group, *n* (*p*%)	182,198,287				0.011
Normal		50,188,044 (27.55%)	48,649,756 (27.80%)	1,538,288 (21.40%)	
Obese		59,645,236 (32.74%)	57,326,996 (32.76%)	2,318,240 (32.24%)	
Overweight		72,365,007 (39.72%)	69,031,772 (39.44%)	3,333,234 (46.36%)	
Drink, *n* (*p*%)	182,198,287				0.003
No		43,031,419 (23.62%)	40,882,807 (23.36%)	2,148,611 (29.88%)	
Yes		139,166,868 (76.38%)	134,125,717 (76.64%)	5,041,151 (70.12%)	
Hypertension, *n* (*p*%)	182,198,287				< 0.001
No		121,267,480 (66.56%)	117,959,723 (67.40%)	3,307,757 (46.01%)	
Yes		60,930,806 (33.44%)	57,048,801 (32.60%)	3,882,005 (53.99%)	
Dyslipidemia, *n* (*p*%)	182,198,287				< 0.001
No		117,455,470 (64.47%)	113,903,823 (65.08%)	3,551,647 (49.40%)	
Yes		64,742,817 (35.53%)	61,104,701 (34.92%)	3,638,116 (50.60%)	
Diabetes, *n* (*p*%)	182,198,287				< 0.001
No		161,983,039 (88.90%)	156,274,370 (89.30%)	5,708,668 (79.40%)	
Yes		20,215,248 (11.10%)	18,734,154 (10.70%)	1,481,094 (20.60%)	
PA, *n* (*p*%)	182,198,287				< 0.001
No		95,787,773 (52.57%)	91,380,923 (52.22%)	4,406,850 (61.29%)	
Yes		86,410,514 (47.43%)	83,627,601 (47.78%)	2,782,912 (38.71%)	
Smoke, *n* (*p*%)	182,198,287				< 0.001
No		103,468,791 (56.79%)	100,282,926 (57.30%)	3,185,864 (44.31%)	
Yes		78,729,496 (43.21%)	74,725,598 (42.70%)	4,003,898 (55.69%)	
LC9, *n* (*p*%)	182,198,287				< 0.001
Q1		37,949,851 (20.83%)	35,312,691 (20.18%)	2,637,160 (36.68%)	
Q2		42,737,956 (23.46%)	40,988,722 (23.42%)	1,749,234 (24.33%)	
Q3		47,673,820 (26.17%)	45,986,898 (26.28%)	1,686,922 (23.46%)	
Q4		53,836,660 (29.55%)	52,720,213 (30.12%)	1,116,447 (15.53%)	

### 3.2 Association Between LC9 Score and Risk of Rheumatoid Arthritis

Logistic regression models were applied to evaluate the association between LC9 score and the risk of RA (Table 2). When treated as a continuous variable, each unit increase in LC9 was significantly associated with a reduced odds of RA, with odds ratios (ORs) of 0.60 (95% CI: 0.56–0.65) in Model 1, 0.71 (95% CI: 0.66–0.77) in Model 2, and 0.73 (95% CI: 0.68–0.80) in Model 3 (all *p* < 0.0001). In the quartile‐based analyses, using Q1 (lowest LC9 level) as the reference, the ORs for Q2, Q3, and Q4 in the fully adjusted Model 3 were 0.74 (95% CI: 0.62–0.89), 0.65 (95% CI: 0.53–0.79), and 0.43 (95% CI: 0.33–0.55), respectively (all *p* < 0.001), indicating a clear dose–response relationship. The P for trend remained highly significant across all models (*p* < 0.0001). These findings suggest that higher LC9 scores are strongly and independently associated with a lower risk of RA, highlighting the potential protective role of ideal cardiovascular and psychosocial health in RA prevention.

**TABLE 2:** Weighted logistic regression of LC9 and RA risk across models.

Variable	OR (95% CI), *p*‐value		
	Model 1	Model 2	Model 3
LC9	0.60 (0.56, 0.65) < 0.0001	0.71 (0.66, 0.77) < 0.0001	0.73 (0.68, 0.80) < 0.0001
Stratified by LC9 quartiles			
Q1	Reference	Reference	Reference
Q2	0.63 (0.53, 0.75)	0.72 (0.60, 0.85)	0.74 (0.62, 0.89)
	< 0.0001	0.0002	0.0009
Q3	0.46 (0.38, 0.56)	0.611 (0.50, 0.74)	0.65 (0.53, 0.79) < 0.0001
	< 0.0001	< 0.0001	
Q4	0.23 (0.18, 0.29)	0.39 (0.30, 0.50)	0.43 (0.33, 0.55) < 0.0001
	< 0.0001	< 0.0001	
*p* for trend	< 0.0001	< 0.0001	< 0.0001

*Note:* Model 1: unadjusted; Model 2: adjusted for age, gender, race, marital status, and education level; Model 3: further adjusted for ALT, AST, HDL, PIR, and alcohol consumption based on Model 2. All estimates are weighted based on the NHANES complex survey design. *p* for trend evaluates the linear trend across LC9 quartiles.

### 3.3 Nonlinear Association Between LC9 and Risk of RA

**FIGURE 1:**
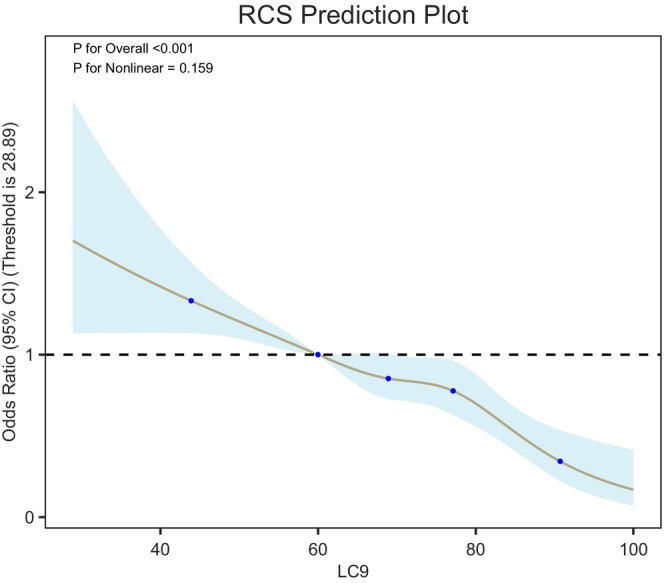
Association between LC9 and RA risk using restricted cubic spline. The plot was generated from a logistic regression model with restricted cubic splines. The reference point was set at 28.89. Shaded area indicates the 95% confidence interval.

Restricted cubic spline (RCS) analysis was conducted to explore the dose–response relationship between LC9 scores and the risk of RA. As shown in Figure 1, the odds of RA decreased significantly with increasing LC9 levels (*p* < 0.001). The test for nonlinearity yielded a *p*‐value of 0.159, indicating that the association followed a linear trend, with no significant evidence of nonlinearity.

### 3.4 Subgroup Analyses

**FIGURE 2:**
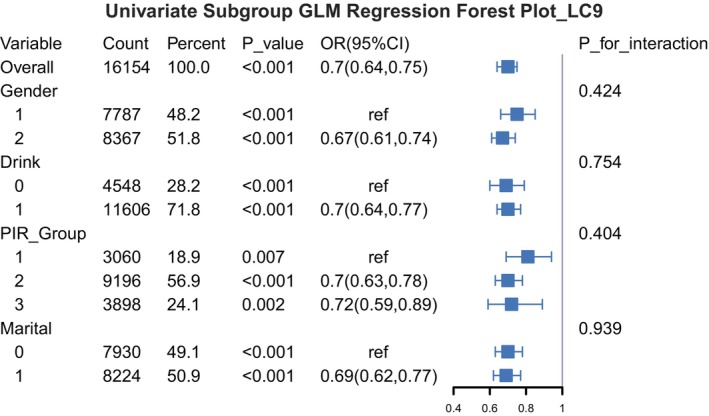
Subgroup analysis of the association between LC9 and RA risk. Weighted univariate logistic regression was performed to estimate ORs and 95% CIs across subgroups. No significant interactions were found (*p* for interaction > 0.05).

To assess the robustness of the association between LC9 and rheumatoid arthritis (RA), weighted univariable logistic regression models were applied across subpopulations defined by demographic characteristics, based on the complex survey design of NHANES. As shown in Figure 2, LC9 was consistently and inversely associated with RA risk across all subgroups—stratified by sex, alcohol consumption status, poverty income ratio (PIR), and marital status—with no significant interactions observed. These findings suggest that the potential protective effect of LC9 against RA is broadly applicable across diverse population strata.

### 3.5 Development and Validation of Disease Prediction Models

**FIGURE 3:**
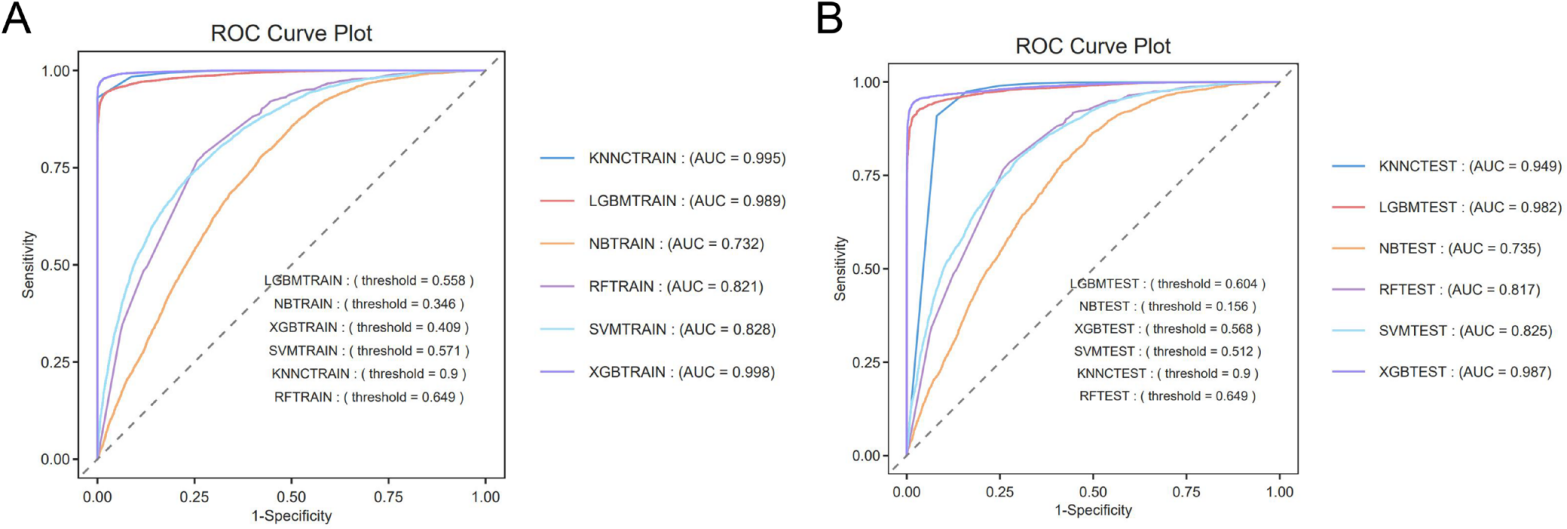
ROC curves of machine learning models in training and testing sets. (A) ROC curves for six machine learning models in the training set. (B) ROC curves for the same models in the testing set.

On the training dataset, machine learning models demonstrated varying degrees of predictive performance. Among them, the eXtreme Gradient Boosting (XGBoost) algorithm exhibited the most outstanding performance, achieving an accuracy of 98.0%, an F1‐score of 0.9801, a Matthews correlation coefficient (MCC) of 0.9611, and an area under the receiver operating characteristic curve (AUROC) of 0.9981‐indicating exceptional discriminative and calibration capabilities (Figure 3A; Table 3). Its robustness was further confirmed across precision‐recall (PR) curves, decision curve analysis (DCA), and Q‐Q plots (Figures S1 and S2).

**TABLE 3:** Comparison of classification performance of different machine learning models on the train set.

Model name	Accuracy	Recall	F1‐Score	MCC	AUROC	Presicion	Specificity	FNR	FPR
RFTRAIN	0.7354	0.8915	0.7710	0.4956	0.8207	0.6793	0.5793	0.1085	0.4207
KNNCTRAIN	0.9047	0.9950	0.9126	0.8229	0.9948	0.8427	0.8145	0.0050	0.1855
SVMTRAIN	0.7359	0.8547	0.7639	0.4858	0.8283	0.6905	0.6172	0.1453	0.3828
XGBTRAIN	0.9804	0.9659	0.9801	0.9611	0.9981	0.9947	0.9949	0.0341	0.0051
NBTRAIN	0.6710	0.7161	0.6851	0.3435	0.7321	0.6567	0.6260	0.2839	0.3740
LGBMTRAIN	0.9593	0.9467	0.9588	0.9189	0.9892	0.9711	0.9718	0.0533	0.0282

The Light Gradient Boosting Machine (LightGBM) model ranked second, with an accuracy of 95.9%, AUROC of 0.9892, and F1‐score and MCC values comparable to those of XGBoost, indicating favorable predictive performance (Figure 3A; Table 3). The K‐nearest neighbors (KNN) model also yielded a high AUROC (0.9948), but exhibited lower precision and PR‐AUC, with overly polarized prediction distributions, reflecting limited stability (Figures S1 and S2).

In contrast, both the support vector machine (SVM) and random forest (RF) models delivered moderate performance across metrics, offering balanced but less distinctive predictive capabilities (Figure 3A; Table 3). The naïve Bayes (NB) model underperformed in all evaluated metrics—including accuracy, F1‐score, AUROC, and calibration reliability—highlighting its limited suitability for this task (Figure 3A; Table 3).

Performance on the independent test set indicated overall good generalizability across models. Both XGBoost and LightGBM maintained superior predictive capabilities, achieving consistently high accuracy and AUROC values, thereby validating their advantages observed in the training phase. The K‐nearest neighbors (KNN) model demonstrated a relatively high recall but lower precision, while the support vector machine (SVM) and random forest (RF) models showed moderate and balanced performance. In contrast, the naïve Bayes (NB) model performed comparatively poorly across key evaluation metrics (Figure 3B; Table 4). Supplementary analyses based on precision‐recall (PR) curves, decision curve analysis (DCA), and Q‐Q plots further corroborated the robustness and practical utility of XGBoost and LightGBM (Figures S3 and S4).

**TABLE 4:** Comparison of classification performance of different machine learning models on the test set.

Model name	Accuracy	Recall	F1‐Score	MCC	AUROC	Presicion	Specificity	FNR	FPR
RFTEST	0.7341	0.8922	0.7705	0.4933	0.8165	0.6781	0.5758	0.1078	0.4242
KNNCTEST	0.8711	0.9898	0.8848	0.7639	0.9486	0.8	0.7522	0.0102	0.2478
SVMTEST	0.7396	0.8563	0.7670	0.4928	0.8252	0.6945	0.6228	0.1437	0.3772
XGBTEST	0.9620	0.9506	0.9616	0.9243	0.9872	0.9729	0.9734	0.0494	0.0266
NBTEST	0.6728	0.7121	0.6854	0.3467	0.7353	0.6605	0.6335	0.2879	0.3665
LGBMTEST	0.9412	0.9341	0.9409	0.8826	0.9816	0.9477	0.9484	0.0659	0.0516

To minimize potential bias arising from data partitioning between training and test sets, fivefold cross‐validation was employed to assess the stability and generalizability of the XGBoost model. As shown in Table S2, the model achieved AUC values ranging from 0.9856 to 0.9891 across the five folds (mean AUC = 0.9875), consistently demonstrating high classification performance and strong robustness (Figures S5 and S6; Table S2). Taken together, these findings establish XGBoost as the most performant and reliable classification algorithm in the present study.

### 3.6 Feature Importance of LC9 Based on SHAP Analysis

**FIGURE 4:**
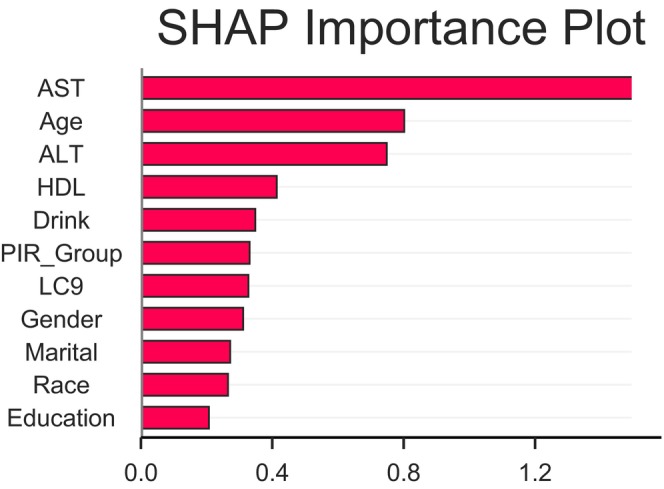
SHAP‐based feature importance plot.

As illustrated in Figure 4, SHAP value analysis revealed that LC9 exhibited a moderate level of feature importance within the predictive model. While its contribution was lower than that of AST and age, it was comparable to variables such as alcohol consumption and poverty income ratio (PIR group), underscoring its independent predictive value. SHAP values at the individual level showed variability, with LC9 exerting a notable influence on prediction outcomes in certain participants, suggesting potential discriminative utility within specific subpopulations. Overall, LC9 maintained a consistent and moderate predictive weight in the model.

## 4 Discussion

This study, based on large‐scale cross‐sectional data from NHANES, is the first to systematically evaluate the association between the comprehensive cardiovascular health index LC9 score and the risk of RA. The analysis revealed a significant negative correlation between LC9 scores and RA risk. In the multivariable binary logistic regression model (Table 2, Model 3), each 1‐unit increase in LC9 score was associated with a 27% reduction in RA risk (OR = 0.73, 95% CI: 0.68–0.80, *p* < 0.0001). When stratified by LC9 quartiles, individuals in the highest quartile (Q4) had a 57% lower risk of RA compared to those in the lowest quartile (Q1) (OR = 0.43, 95% CI: 0.33–0.55, *p* < 0.0001), with a significant dose–response relationship (trend *p* < 0.0001). Restricted cubic spline analysis (Figure 1) further confirmed a significant linear negative correlation between LC9 score and RA risk (*p* < 0.001), with no evidence of nonlinearity (*p* = 0.159).

For risk prediction, this study innovatively employed machine learning techniques. An XGBoost‐based predictive model was constructed by integrating LC9 scores with multiple covariates. In the independent test set, the model demonstrated excellent predictive performance, with an area under the receiver operating characteristic curve (AUROC) as high as 0.987 (Table 4), significantly outperforming other comparative models (e.g., random forest AUROC = 0.8165). These findings underscore the practical value of LC9 in RA risk stratification and provide robust epidemiological evidence for the role of cardiometabolic‐immune homeostasis in the pathogenesis of RA, establishing LC9 as a quantifiable and modifiable multidimensional health index that may serve as a potential predictor of RA risk.

The study further found that RA patients generally exhibited poorer cardiometabolic and mental health status (Table 1), with significantly lower LC9 scores compared to non‐RA individuals (weighted mean: 63.38 vs. 70.31, *p* < 0.001). These health disparities may influence RA risk through multiple pathological mechanisms. For example, the RA group had a higher mean body mass index (BMI: 30.71 vs. 29.38) and a greater proportion of smokers (55.69% vs. 42.70%), consistent with known mechanisms. Previous research has shown that in obesity, adipose tissue releases leptin and free fatty acids that can activate synovial inflammation, promote the release of pro‐inflammatory cytokines, and exacerbate arthritis (Song et al. 2020; Frommer et al. 2019). Wouters et al. (Wouters et al. 2022) further suggested that components of tobacco can promote the production of anti‐citrullinated protein antibodies (ACPA) and seroconversion, triggering early autoimmune responses.

Although LDL levels did not differ significantly between RA and non‐RA groups (weighted mean: 112.97 vs. 112.58 mg/dL, *p* = 0.879), the prevalence of dyslipidemia was substantially higher in RA patients (50.60% vs. 34.92%). Together with previous studies (Popova et al. 2022; Weber et al. 2023), this suggests that inflammatory mediators such as TNF‐α and IL‐6 may drive synovial inflammation, bone destruction, and atherosclerosis progression through shared pathways such as JAK–STAT. Imaging studies also confirm that RA patients exhibit a significantly higher coronary non‐calcified plaque burden, with conventional cardiovascular risk models often underestimating their risk (Karpouzas et al. 2021).

In addition, fasting plasma glucose levels were significantly higher in the RA group compared with controls (FPG: 112.27 vs. 109.19 mg/dL). Hyperglycemia may promote bone erosion by enhancing osteoclast activity through advanced glycation end products (AGEs) (Cui et al. 2011). Metabolic studies by Arias de la Rosa (Arias de la Rosa et al. 2018) revealed that hyperglycemia and chronic inflammation form a vicious cycle: TNF‐α in RA patients inhibits IRS‐1 phosphorylation in adipose tissue, inducing insulin resistance, which in turn promotes IL‐6 release. Clinical evidence from Charoenngam (Charoenngam et al. 2021) further supports the direct influence of glycemic regulation on RA risk, as DPP‐4 inhibitor therapy reduced RA incidence by 28% in patients with type 2 diabetes.

Moreover, RA patients exhibited significantly lower participation in physical activity (38.71% vs. 47.78%), and their lower LC9 psychological health scores are consistent with the theory that chronic stress exacerbates systemic inflammation via neuroendocrine and immune pathways. Nerurkar et al. (Nerurkar et al. 2019) reported that increased depression prevalence in RA patients is linked to pro‐inflammatory cytokine‐mediated neural plasticity damage. Stanciu et al. (Stanciu et al. 2022) further confirmed that RA patients are at higher risk of insomnia and morning fatigue, while Matcham's systematic review (Matcham et al. 2018) emphasized the limited effectiveness of pharmacological treatment alone in improving psychological health. These findings align with the observed reduction in LC9 psychological health scores in this study.

In summary, the nine dimensions encompassed by the LC9 score, particularly obesity, smoking, hyperglycemia, physical inactivity, and mental health, jointly constitute potential pathophysiological foundations underlying the development of RA.

The primary advantage of the XGBoost model constructed in this study lies in its ability to effectively capture and learn the complex nonlinear associations and interactions among multidimensional health data. The algorithm's adaptive optimization mechanism enables the integration of composite cardiovascular health indicators, such as LC9, with diverse heterogeneous covariates in risk prediction. Through SHAP interpretability analysis, the stable contribution of the LC9 score as an independent predictor was confirmed. Its importance ranked at a moderate level, lower than key variables such as AST and age, but higher than sociobehavioral factors such as alcohol consumption and PIR grouping. The analysis also revealed the potential predictive value of covariates that might otherwise be overlooked in conventional statistical models, providing new insights for exploring their biological mechanisms. Therefore, integrating LC9 with machine learning prediction models offers a high‐performance and interpretable new tool for early warning and effective stratification of high‐risk populations with RA. This methodological advancement significantly enhances predictive performance. Moreover, the results further strengthen, from a data‐driven perspective, the crucial role of improving cardiovascular health in reducing RA risk, thus laying a methodological foundation for developing targeted prevention strategies. However, due to the low proportion of RA‐positive samples in the test set, the model's high accuracy should be evaluated in conjunction with other indicators such as sensitivity.

The XGBoost model achieved an AUC of 0.987 on the test set. This level of performance may be explained by several aspects of the study design and data characteristics. The LC9 score encompasses nine health dimensions that are directly linked to RA pathogenesis, including obesity, smoking, blood glucose, and mental health, as discussed earlier. When laboratory markers such as AST, ALT, and HDL were included alongside LC9, the combined feature set might have captured a broad spectrum of RA‐related systemic inflammation. Moreover, the five‐fold cross‐validation results, presented in Table S2, demonstrated consistently high AUC values across the five folds, with a mean of 0.9875 and a range from 0.9856 to 0.9891. This consistency suggests that the high predictive performance was not merely due to overfitting or random variation. The training set AUC (0.998, as shown in Table 3) was very close to the test set AUC (0.987), further supporting the model's generalizability.

Several limitations should be considered when interpreting this high AUC. First, the cross‐sectional nature of the NHANES data precludes causal inference; the observed association between LC9 and RA does not confirm that improving LC9 reduces RA risk. Second, RA status was determined by self‐report, which may introduce misclassification bias. Third, although cross‐validation indicated stability, the slight drop from training AUC (0.998) to test AUC (0.987) suggests possible mild overfitting. Fourth, the model was developed and tested on the same NHANES population without external validation in an independent RA cohort. Therefore, the reported AUC of 0.987 likely represents an upper‐bound estimate, and external validation is needed before clinical application.

In summary, this study, for the first time in a large representative population, systematically revealed a significant negative association between LC9 and the risk of RA, with a clear dose–response relationship and linear trend. This finding reinforces the critical role of cardiovascular‐metabolic‐immune homeostasis in the pathogenesis of RA. At the same time, it establishes LC9, a modifiable, quantifiable, and multidimensional health indicator system, as a potential predictor of RA risk. This provides a novel scientific basis for moving beyond the traditional single risk factor approach toward comprehensive health risk assessment to identify high‐risk populations for RA. Subgroup analyses further confirmed the universality of this protective association across different demographic groups, particularly among populations with lower socioeconomic status, highlighting the potential of optimizing cardiovascular health as a broadly applicable preventive strategy against RA. However, this study has several limitations. First, although the cross‐sectional design strongly reveals the association between LC9 scores and RA, it cannot establish causality or determine temporal sequence. It is therefore difficult to assess whether poor cardiovascular health is a cause or consequence of RA, or whether both are driven by shared factors. Second, reliance on self‐reported RA diagnoses carries a risk of misclassification, potentially introducing information bias. Third, physical activity was not included as an independent covariate. Physical activity is one of the nine dimensions of the LC9 score, and its level may have a substantial impact on the study results. Previous studies have shown that regular aerobic or resistance exercise can improve clinical symptoms and reduce the risk of cardiovascular disease in patients with rheumatoid arthritis (Metsios et al. 2020; Coskun Benlidayi 2024). The cardiovascular protective effects of exercise are primarily achieved through improved vascular function, reduced systemic inflammation, restored autonomic balance, and regulated lipid metabolism (Coskun Benlidayi 2024). These findings indicate that physical activity plays an important role in the prevention of cardiovascular disease among RA patients. In the present study, physical activity was not included as a separate variable because it was already incorporated into the LC9 score. Therefore, the independent effect of physical activity on RA risk could not be assessed. As a result, it remains unclear whether the observed reduction in RA risk is attributable to physical activity itself or to the combined influence of other LC9 dimensions. Future studies using more precise measurements of physical activity (e.g., accelerometers or detailed questionnaires) and including physical activity as an independent variable will help to further clarify this issue. Finally, the study did not account for the possible confounding effects of antirheumatic, cardiovascular, or other therapeutic medications on LC9 components and their association with RA. Based on these findings and limitations, future studies should employ prospective cohort designs to clarify the causal relationship between LC9 and RA, and conduct intervention trials to verify the effectiveness of optimizing cardiovascular health in RA prevention.

## Author Contributions

**Jiasi Zheng:** writing – original draft. **Zhen Wang:** software. **Wukai Ma:** writing – review and editing. **Ziyuan Song:** validation, investigation. **Xuemei Yuan:** methodology. **Yuanyuan Gao:** conceptualization. **Feng Luo:** data curation.

## Funding

National Natural Science Foundation of China (82274678).

## Ethics Statement

The data used in this study were obtained from the National Health and Nutrition Examination Survey (NHANES), which was approved by the National Center for Health Statistics (NCHS) Ethics Review Board, and all participants provided written informed consent. This study is a secondary analysis of de‐identified publicly available data and is exempt from additional institutional review board approval.

## Consent

The authors have nothing to report.

## Conflicts of Interest

The authors declare no conflicts of interest.

## Supporting information


**Table S1:** Unweighted baseline characteristics of the study population.
**Table S2:** Five‐fold cross‐validation performance metrics of the XGBoost model.
**Figure S1:** Calibration plot, decision curve analysis (DCA), and precision‐recall (PR) curve for the training set.
**Figure S2:** Q‐Q plots of predicted probabilities for the training set.
**Figure S3:** Calibration plot, DCA, and PR curve for the test set.
**Figure S4:** Q‐Q plots of predicted probabilities for the test set.
**Figure S5:** Five‐fold cross‐validation evaluation of the XGBoost model (calibration, DCA, PR, ROC).
**Figure S6:** Q‐Q plots for the five XGBoost test models.

## Data Availability

The datasets analyzed in this study are publicly available from the NHANES website: https://www.cdc.gov/nchs/nhanes/.
