# Supplementary material for: Utilizing Life's Crucial 9 for Rheumatoid Arthritis Risk Prediction: A Machine Learning Approach Based on NHANES Data
Source: Food Sci Nutr. 2026 May 21;14(5):e71931. doi: 10.1002/fsn3.71931 (PMC13240368; doi:10.1002/fsn3.71931)
Supplement: Supplementary file 1 — Table S1: Unweighted baseline characteristics of the study population. Table S2: Five‐fold cross‐validation performance metrics of the XGBoost model. Figure S1: Calibration plot, decision curve analysis (DCA), and precision‐recall (PR) curve for the training set. Figure S2: Q‐Q plots of predicted probabilities for the training set. Figure S3: Calibration plot, DCA, and PR curve for the test set. Figure S4: Q‐Q plots of predicted probabilities for the test set. Figure S5: Five‐fold cross‐validation evaluation of the XGBoost model (calibration, DCA, PR, ROC). Figure S6: Q‐Q plots for the five XGBoost test models. [file FSN3-14-e71931-s001.docx]

**Table S1. Unweighted Descriptive Statistics of Baseline Characteristics**

|  |  | RA | |  |
| --- | --- | --- | --- | --- |
| Variable | Overall | No | Yes | *p*-value |
|  | N = 16,154 | N = 15,318 | N = 836 |  |
| Age, mean (sd) | 49.99 (17.54) | 49.40 (17.58) | 60.69 (12.88) | <0.001 |
| PIR, mean (sd) | 2.55 (1.56) | 2.57 (1.56) | 2.15 (1.45) | <0.001 |
| BMI, mean (sd) | 29.58 (7.15) | 29.49 (7.11) | 31.24 (7.64) | <0.001 |
| SBP, mean (sd) | 124.19 (17.12) | 123.90 (16.99) | 129.54 (18.58) | <0.001 |
| DBP, mean (sd) | 70.30 (11.85) | 70.34 (11.73) | 69.50 (13.93) | 0.088 |
| AST, mean (sd) | 24.79 (17.04) | 24.76 (17.08) | 25.46 (16.34) | 0.231 |
| ALT, mean (sd) | 24.46 (20.61) | 24.50 (20.88) | 23.61 (14.75) | 0.098 |
| FPG, mean (sd) | 110.63 (24.61) | 110.46 (24.50) | 113.63 (26.36) | <0.001 |
| HDL, mean (sd) | 53.17 (15.68) | 53.14 (15.66) | 53.72 (16.04) | 0.302 |
| TC, mean (sd) | 190.64 (41.06) | 190.67 (41.08) | 190.07 (40.81) | 0.677 |
| TG, mean (sd) | 119.26 (72.61) | 119.27 (73.68) | 119.07 (48.93) | 0.909 |
| LDL, mean (sd) | 112.24 (23.96) | 112.30 (23.97) | 111.13 (23.85) | 0.166 |
| LC9, mean (sd) | 68.16 (14.04) | 68.53 (13.99) | 61.32 (13.33) | <0.001 |
| Gender, n (p%) |  |  |  | 0.001 |
| Male | 7,787.00 (48.20%) | 7,430.00 (48.51%) | 357.00 (42.70%) |  |
| Female | 8,367.00 (51.80%) | 7,888.00 (51.49%) | 479.00 (57.30%) |  |
| Race, n (p%) |  |  |  | <0.001 |
| Mexican American | 2,074.00 (12.84%) | 1,964.00 (12.82%) | 110.00 (13.16%) |  |
| Non-Hispanic Black | 1,610.00 (9.97%) | 1,527.00 (9.97%) | 83.00 (9.93%) |  |
| Non-Hispanic White | 6,398.00 (39.61%) | 6,093.00 (39.78%) | 305.00 (36.48%) |  |
| Other Hispanic | 3,742.00 (23.16%) | 3,473.00 (22.67%) | 269.00 (32.18%) |  |
| Other race | 2,330.00 (14.42%) | 2,261.00 (14.76%) | 69.00 (8.25%) |  |
| Education, n (p%) |  |  |  | <0.001 |
| Above high school | 1,915.00 (11.85%) | 1,786.00 (11.66%) | 129.00 (15.43%) |  |
| High school | 3,627.00 (22.45%) | 3,425.00 (22.36%) | 202.00 (24.16%) |  |
| Under high school | 10,612.00 (65.69%) | 10,107.00 (65.98%) | 505.00 (60.41%) |  |
| Marital, n (p%) |  |  |  | 0.495 |
| Living alone | 7,930.00 (49.09%) | 7,510.00 (49.03%) | 420.00 (50.24%) |  |
| Living with a partner | 8,224.00 (50.91%) | 7,808.00 (50.97%) | 416.00 (49.76%) |  |
| PIR_Group, n (p%) |  |  |  | <0.001 |
| <1.30 | 3,060.00 (18.94%) | 2,834.00 (18.50%) | 226.00 (27.03%) |  |
| 1.30~3.49 | 9,196.00 (56.93%) | 8,719.00 (56.92%) | 477.00 (57.06%) |  |
| ≥3.50 | 3,898.00 (24.13%) | 3,765.00 (24.58%) | 133.00 (15.91%) |  |
| BMI_Group, n (p%) |  |  |  | <0.001 |
| Normal | 4,382.00 (27.13%) | 4,230.00 (27.61%) | 152.00 (18.18%) |  |
| Obese | 5,278.00 (32.67%) | 5,000.00 (32.64%) | 278.00 (33.25%) |  |
| Overweight | 6,494.00 (40.20%) | 6,088.00 (39.74%) | 406.00 (48.56%) |  |
| Drink, n (p%) |  |  |  | 0.001 |
| No | 4,548.00 (28.15%) | 4,271.00 (27.88%) | 277.00 (33.13%) |  |
| Yes | 11,606.00 (71.85%) | 11,047.00 (72.12%) | 559.00 (66.87%) |  |
| Hypertension, n (p%) |  |  |  | <0.001 |
| No | 10,053.00 (62.23%) | 9,737.00 (63.57%) | 316.00 (37.80%) |  |
| Yes | 6,101.00 (37.77%) | 5,581.00 (36.43%) | 520.00 (62.20%) |  |
| Dyslipidemia, n (p%) |  |  |  | <0.001 |
| No | 10,298.00 (63.75%) | 9,906.00 (64.67%) | 392.00 (46.89%) |  |
| Yes | 5,856.00 (36.25%) | 5,412.00 (35.33%) | 444.00 (53.11%) |  |
| Diabetes, n (p%) |  |  |  | <0.001 |
| No | 13,804.00 (85.45%) | 13,201.00 (86.18%) | 603.00 (72.13%) |  |
| Yes | 2,350.00 (14.55%) | 2,117.00 (13.82%) | 233.00 (27.87%) |  |
| PA, n (p%) |  |  |  | <0.001 |
| No | 9,324.00 (57.72%) | 8,770.00 (57.25%) | 554.00 (66.27%) |  |
| Yes | 6,830.00 (42.28%) | 6,548.00 (42.75%) | 282.00 (33.73%) |  |
| Smoke, n (p%) |  |  |  | <0.001 |
| No | 9,227.00 (57.12%) | 8,839.00 (57.70%) | 388.00 (46.41%) |  |
| Yes | 6,927.00 (42.88%) | 6,479.00 (42.30%) | 448.00 (53.59%) |  |
| LC9, n (p%) |  |  |  | <0.001 |
| Q1 | 4,038.00 (25.00%) | 3,687.00 (24.07%) | 351.00 (41.99%) |  |
| Q2 | 4,038.00 (25.00%) | 3,809.00 (24.87%) | 229.00 (27.39%) |  |
| Q3 | 4,038.00 (25.00%) | 3,869.00 (25.26%) | 169.00 (20.22%) |  |
| Q4 | 4,040.00 (25.01%) | 3,953.00 (25.81%) | 87.00 (10.41%) |  |

**Table S2. Performance Metrics of Five-Fold Cross-Validation Using XGBoost Models**

| ModelName | Accuracy | Prevalence | Recall | F1-Score | MCC | AUROC | Presicion | Specificity | FNR | FPR |
| --- | --- | --- | --- | --- | --- | --- | --- | --- | --- | --- |
| XGB_1TEST | 0.9659 | 0.4953 | 0.9483 | 0.9650 | 0.9323 | 0.9891 | 0.9823 | 0.9832 | 0.0517 | 0.0168 |
| XGB_2TEST | 0.9633 | 0.4885 | 0.9462 | 0.9618 | 0.9269 | 0.9864 | 0.9779 | 0.9796 | 0.0538 | 0.0204 |
| XGB_3TEST | 0.9594 | 0.5043 | 0.9456 | 0.9591 | 0.9191 | 0.9856 | 0.9730 | 0.9733 | 0.0544 | 0.0267 |
| XGB_4TEST | 0.9670 | 0.5107 | 0.9556 | 0.9673 | 0.9344 | 0.9886 | 0.9794 | 0.9790 | 0.0444 | 0.0210 |
| XGB_5TEST | 0.9634 | 0.5011 | 0.9459 | 0.9629 | 0.9275 | 0.9878 | 0.9804 | 0.9810 | 0.0541 | 0.0190 |
| mean_scores | 0.9638 | 0.5 | 0.9483 | 0.9632 | 0.928 | 0.9875 | 0.9786 | 0.9792 | 0.0517 | 0.0208 |


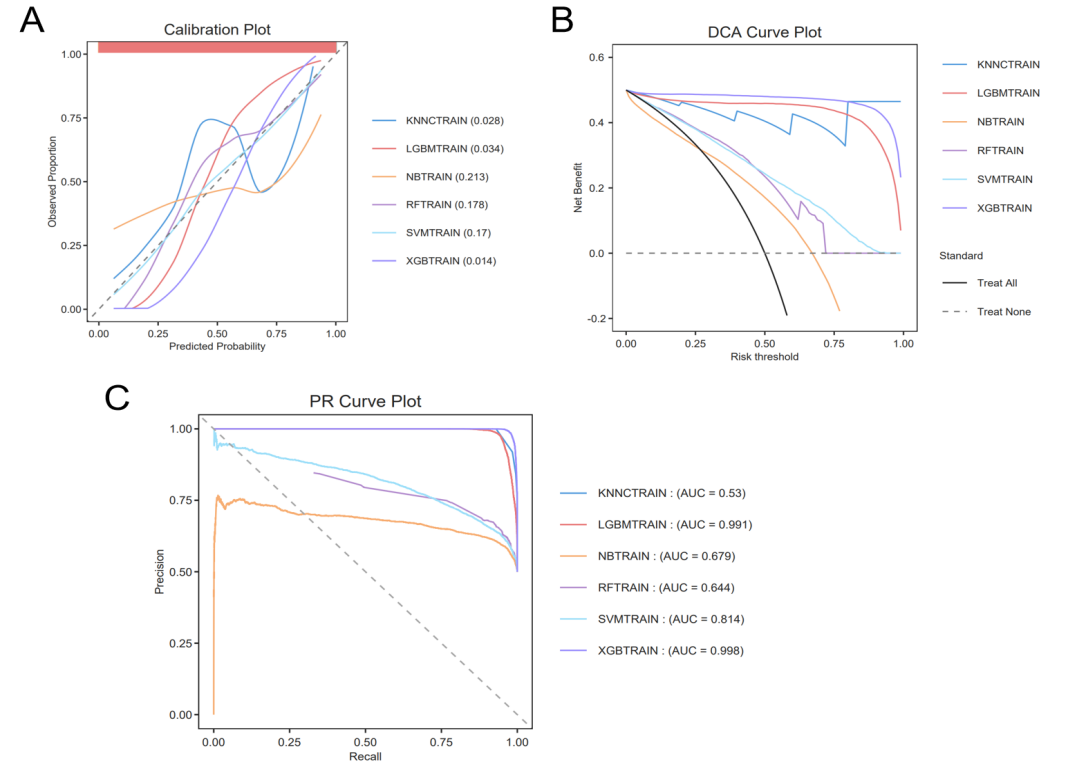


**Fig S1. Comprehensive performance evaluation of models in the training set**

**Note:**A. Calibration plot: Assesses the agreement between predicted probabilities and observed outcomes for each model. B. Decision curve analysis (DCA): Shows net clinical benefit across a range of risk thresholds. C. Precision-recall (PR) curve: Illustrates the trade-off between precision and recall for each model.


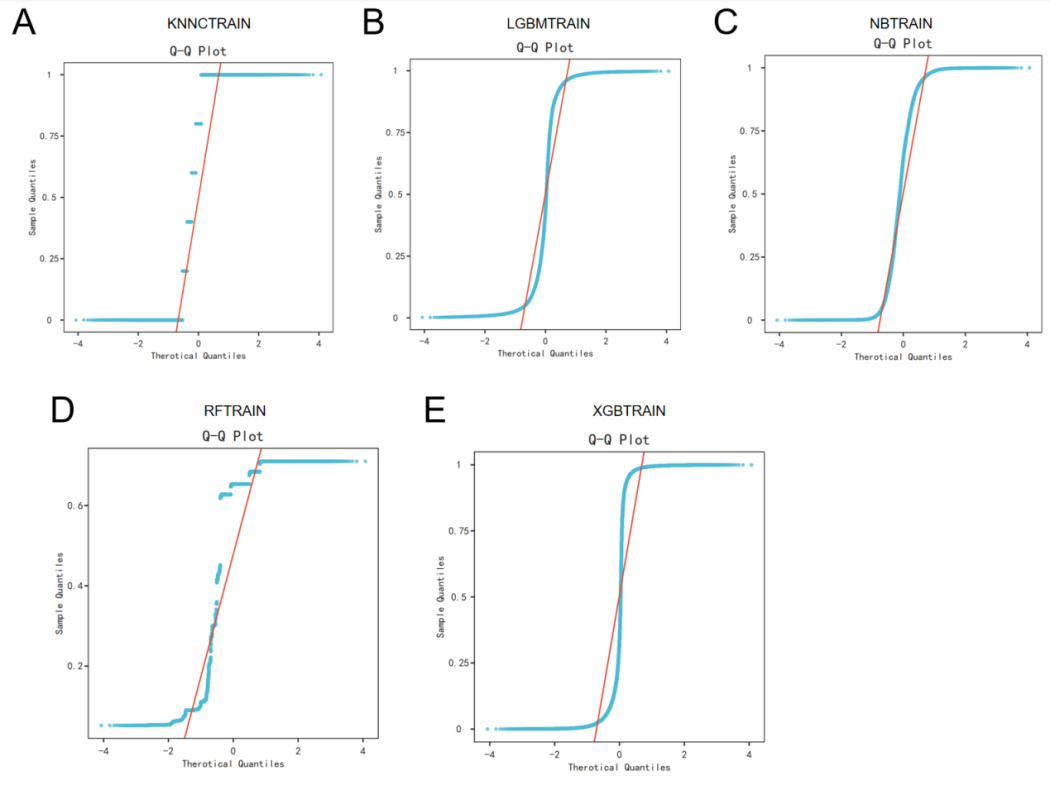


**Fig S2. Q-Q plots of predicted probabilities for models in the training set**

**Note:** Quantile-Quantile (Q-Q) plots comparing the distribution of predicted probabilities with the theoretical distribution, used to assess goodness-of-fit.


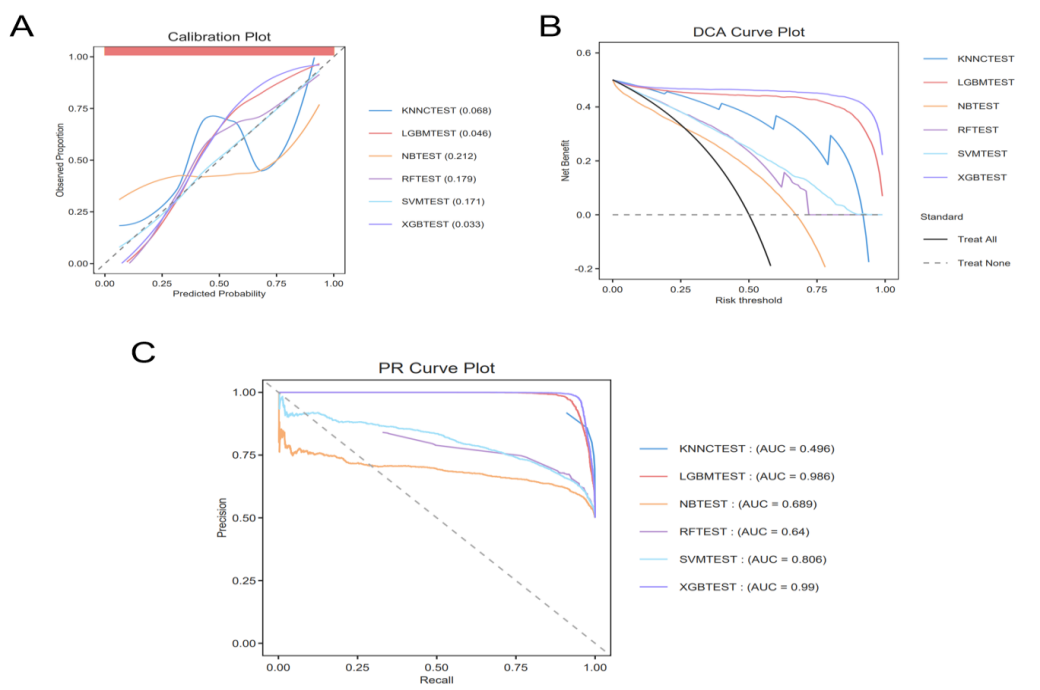


**Fig S3. Calibration, Decision Curve Analysis (DCA), and Precision-Recall (PR) curves of models on the test set**


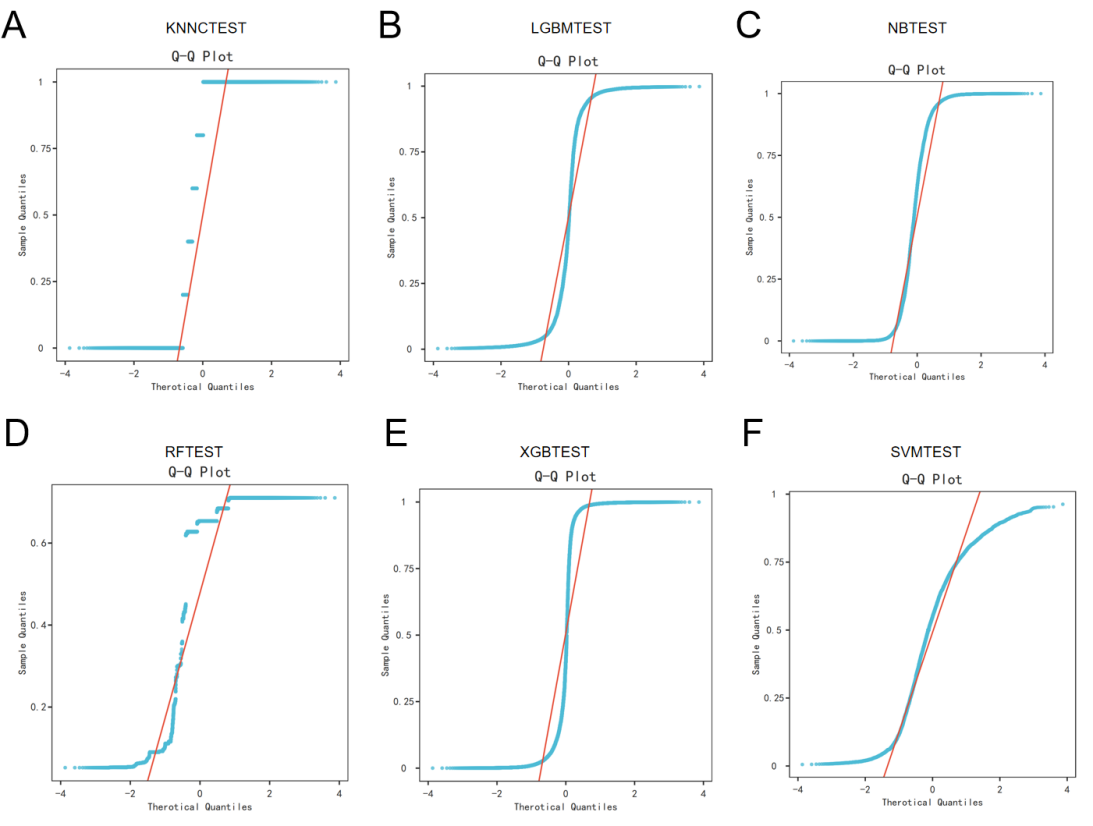


**Fig S4. Q-Q plots of predicted values from each model**

**Note:** Quantile-Quantile (Q-Q) plots comparing the distribution of predicted probabilities with the theoretical distribution, used to assess goodness-of-fit.


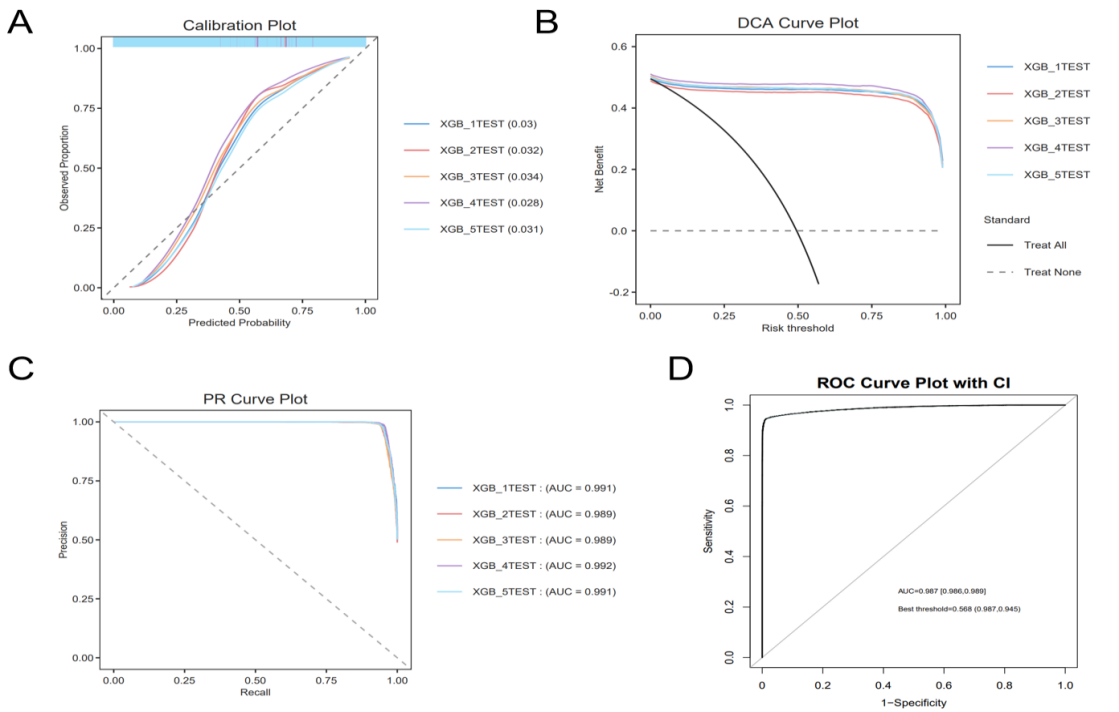


**Fig S5. Evaluation of XGBoost Model Performance Across Five-Fold Cross-Validation**

**Note:** (A) Calibration curves for five XGBoost test sets show agreement between predicted and observed probabilities. (B) Decision curve analysis (DCA) illustrates net benefit across risk thresholds. (C) Precision-recall (PR) curves indicate model accuracy and completeness. (D) ROC curve with 95% confidence interval (CI) confirms strong discriminative ability (AUC=0.987, 95% CI: 0.986-0.989).


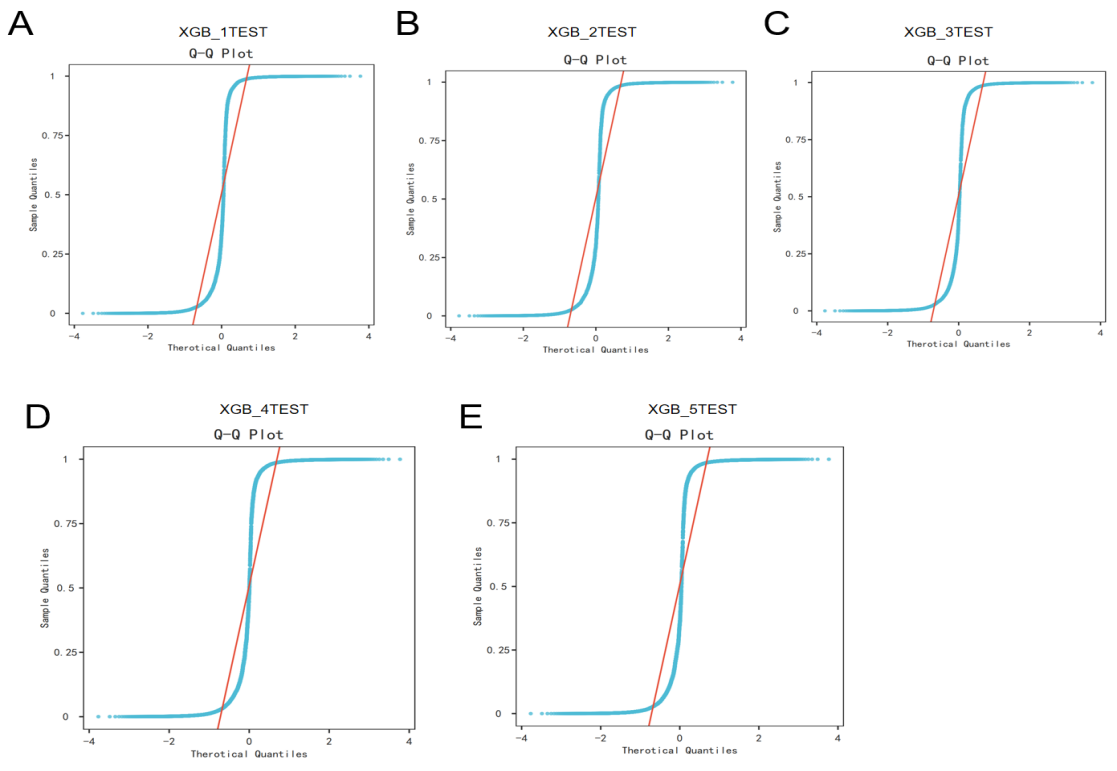


**Fig S6. Q-Q Plots for Five XGBoost Test Models**

**Note:**Each subplot shows the Q-Q plot of predicted probabilities versus theoretical quantiles from five-fold cross-validation XGBoost test sets, indicating the normality of residuals.
